# Using Thermally Crosslinkable Hole Transporting Layer to Improve Interface Characteristics for Perovskite CsPbBr_3_ Quantum-Dot Light-Emitting Diodes

**DOI:** 10.3390/polym12102243

**Published:** 2020-09-29

**Authors:** Chun-Cheng Lin, Shao-Yang Yeh, Wei-Lun Huang, You-Xun Xu, Yan-Siang Huang, Tzu-Hung Yeh, Ching-Ho Tien, Lung-Chien Chen, Zong-Liang Tseng

**Affiliations:** 1 Department of Mathematic and Physical Sciences, General Education, R.O.C. Air Force Academy, Kaohsiung 820008, Taiwan; cclincafa@gmail.com; 2 Department of Electro-Optical Engineering, National Taipei University of Technology, Taipei 10608, Taiwan; iamarvin0811@gmail.com (S.-Y.Y.); chtien@mail.ntut.edu.tw (C.-H.T.); 3 Department of Electronic Engineering, Ming Chi University of Technology, New Taipei City 243303, Taiwan; a88061446@gmail.com (W.-L.H.); sasa220404373@gmail.com (Y.-X.X.); jim824645@gmail.com (Y.-S.H.); 4 Organic Electronics Research Center, Ming Chi University of Technology, New Taipei City 243303, Taiwan; d10502004@mail.ntust.edu.tw

**Keywords:** VB-FNPD, perovskite, CsPbBr_3_ QDs, light-emitting diodes

## Abstract

In this paper, a thermally crosslinkable 9,9-Bis[4-[(4-ethenylphenyl)methoxy]phenyl]-N2,N7-di-1-naphthalenyl-N2,N7-diphenyl-9H-fluorene-2,7-diamine (VB-FNPD) film served as the hole transporting layer (HTL) of perovskite CsPbBr_3_ quantum-dot light-emitting diodes (QD-LEDs) was investigated and reported. The VB-FNPD film crosslinked at various temperatures in the range of 100~230 °C followed by a spin-coating process to improve their chemical bonds in an attempt to resist the erosion from the organic solvent in the remaining fabrication process. It is shown that the device with VB-FNPD HTL crosslinking at 170 °C has the highest luminance of 7702 cd/m^2^, the maximum current density (J) of 41.98 mA/cm^2^, the maximum current efficiency (CE) of 5.45 Cd/A, and the maximum external quantum efficiency (EQE) of 1.64%. Our results confirm that the proposed thermally crosslinkable VB-FNPD is a candidate for the HTL of QD-LEDs.

## 1. Introduction

Under the explosive demand for flat-panel display technologies of internet of things (IoT) and artificial intelligence (AI) generation, organic light-emitting diodes (OLEDs) have attracted extensive attention due to their potential advantages, e.g., self-emissive ability, low operating voltage, low panel thickness, fast response time, high luminance, large spectral range, and large size/flexible capability [1,2,3,4,5]. The most efficient devices of OLEDs reported to date have been multilayer structures, i.e., a hole transporting layer (HTL), an active emitting layer (AEL), and an electron transporting layer (ETL) sandwiched between two electrodes. The superiority of a multilayer structure includes facilitated charge carrier injection through reducing the respective injection barriers, enhanced recombination of electrons and holes in the AEL, and decreased exciton quenching by the electrodes [6]. Multilayer device fabrication is usually implemented by one of the methods: high-vacuum vapor deposition or solution processing. The former was limited to thermally stable low-molecular-weight materials, and relatively expensive and time-consuming. While fabrication of devices by the latter using macromolecules would be an attractive alternative, however, one particular requirement is that each deposited layer should be resistant to the solvent used to deposit subsequent layers [7].

To satisfy consumers’ requirement for a higher resolution of display, the pixel size of OLEDs should be more compact and smaller. Therefore, quantum dots (QDs) with only a few nanometers of particle size are very suitable for the purpose [8,9,10,11]. Among them, perovskite QDs have a high quantum efficiency and narrow emission spectra (i.e., full width at half maximum (FWHM)) due to a low trap density, and thus can express more realistic colors with high color reproducibility [12]. Another advantage of perovskite QDs for display is that the emission-color can be controlled by appropriate adjustment of the QD size, which is favorable for producing full-color-tunable QD-LEDs display [13]. However, it remains a big challenge because of the poor stability of multilayer structures, especially the interface miscibility (during the fabrication process) issues [14].

Several strategies have been developed to overcome the aforementioned issues associated with the solution processing of multilayer structures. Among them, choosing an appropriate HTL is a crucial factor for carrier balance of the multilayer structure [15,16]. Prior to this, poly(bis(4-butypheny)-bis(phenyl)benzidine, poly-TPD) is the most commonly used due to a high hole mobility (1.0 × 10^−4^ cm^2^/V·s) with the highest occupied molecular orbital (HOMO) and lowest unoccupied molecular orbital (LUMO) levels of −5.2 and −2.3 eV, respectively, and a high-quality film surface [17]. However, it may suffer from the erosion by the solvent used to deposit subsequent layers. Crosslinking modification (including the chemical and thermal crosslinking) is a promising alternative to improve the mechanical property (e.g., stiffness and robustness) and chemical stability of a polymeric membrane by forming covalent bonds or relatively short sequences of chemical bonds to join polymer chains together [18]. Compared to the chemical crosslinking, the thermal crosslinking is superior because the thermal rearrangement and crosslinking occur in the chain segments of the polymer to form a stable three-dimensional network structure during modification [19,20,21]. Lin et al. proposed a thermally crosslinkable 9,9-Bis[4-[(4-ethenylphenyl)methoxy]phenyl]-N2,N7-di-1-naphthalenyl-N2,N7-diphenyl-9H-fluorene-2,7-diamine (VB-FNPD) as HTL with approximatively high hole mobility (~10^−4^ cm^2^/V·s), similar HOMO and LUMO levels (−5.3 and −2.3 eV, respectively), and remarkable ambient stability (exposing to air for one month) [22,23]. However, to the best of the authors’ knowledge, the temperature dependence of crosslinking behavior of VB-FNPD as HTL of QD-OLEDs has not been discussed. Furthermore, the device performance between poly-TPD and VB-FNPD as HTL has rarely compared.

Accordingly, the present study evaluates the thermally crosslinkable VB-FNPD film served as the HTL of perovskite CsPbBr_3_ QD-LEDs. It is shown that the device with VB-FNPD HTL crosslinking at 170 °C has the highest luminance, the maximum current density (J), the maximum current efficiency (CE), and the maximum external quantum efficiency (EQE). In general, the results confirm that the proposed thermally crosslinkable VB-FNPD is a candidate for the HTL of QD-LEDs due to a better robustness for erosion from the solvent used to deposit subsequent layers.

## 2. Materials and Methods

Materials: Poly(3,4-ethylenedioxythiophene) polystyrene sulfonate (PEDOT:PSS, Al 4083) was purchased from UMAT Co., Ltd. Poly(4-butylphenyl-diphenyl-amine) (poly-TPD), and the VB-FNPD was purchased from Lumtec Corp. Tris-[1-phenyl-1H-benzimidazole] (TPBi) and lithium fluoride (LiF) were purchased from Echo Chemical Co. Ltd. (Miaoli, Taiwan), and used as received. All other materials and solvents were purchased from Echo Chemical Co. Ltd. (Miaoli, Taiwan), and were used as received.

Synthesis of CsPbBr_3_ QDs: Cesium carbonate (200 mg, 99.99%) was loaded into a 25 mL three-neck flask along with octadecene (ODE) (9mL, 90%) and oleic acid (OA) (0.75 mL, 90%). Then, it was stirred on a hot plate at 150 °C for 30 min to obtain a transparent solution. The reaction solution was prepared by dissolving 0.09 M of PbBr_2_ in 30 mL ODE, 3 mL of OA, and 3 mL octylamine. Then, it was also stirred on a hot plate at 120 °C for 30 min to obtain another transparent solution. After that, a 0.8 mL precursor solution was dripped into the reaction solution by a quickly hot-injection method and then decanted into a glass vial for an ice-water bath to compose the unpurified CsPbBr_3_ QDs solutions.

Washing process: Subsequently, they were decanted into 15 mL of toluene with a centrifugation at 6000 rpm for 15 min to become the CsPbBr_3_ QDs powders. Then, the powders were added into the mixed solutions of ethyl acetate and hexane with a volume ratio of 1:3, and then the purified CsPbBr_3_ QDs green precipitates were collected after a centrifugation at 6000 rpm for 10 min. After that, the precipitates were dissolved into 1 mL of hexane (95%, Echo Chemical Co. Ltd., Miaoli, Taiwan), and then vortexed with an ultrasonic oscillator for 10 min to prepare the purified CsPbBr_3_ QDs solution.

Device fabrication: The patterned indium tin oxide (ITO)-coated glass substrates (7 Ω/cm^2^, Lumtec Corp., New Taipei, Taiwan) were cleaned by deionized water, acetone, and isopropyl alcohol for 30 min, and then treated by O_2_ Plasma cleaning for 10 min to remove the residual organic matters and improved the surface work function. After the O_2_ Plasma treatment, the hole injection layer (HIL) of the perovskite CsPbBr_3_ QD-LED, the poly(3,4-ethylenedioxythiophene) polystyrene sulfonate (PEDOT:PSS, Al 4083) was spin-coated at 8000 rpm for 20 s on the substrate and annealed at 120 °C for 15 min, resulting in a 40 nm thick layer. For the preparation of the hole transporting layer with VB-FNPD dissolved in cyclohexanone with a concentration of 4 mg/mL and then deposited onto the HIL by a spin-coating method at 6000 rpm for 15 s as HTL. After that, the samples were immediately annealed at 100~230 °C for 10 min to proceed crosslink. For comparison, poly-TPD was also dissolved into cyclohexanone (CHO) and then deposited onto the HIL by a spin-coating method at 5000 rpm for 30 s resulted in a 20 nm thick layer. CsPbBr_3_ QDs were spin-coated onto VB-FNPD and poly-TPD at 2000 rpm for 15 s in a N_2_-filled glove box. Finally, a 40-nm-thick TPBi as ETL, a 1-nm-thick LiF buffer layer, and a 100-nm-thick Al cathode were deposited using a thermal evaporation system at rates of 0.5~0.6, 0.02~0.03, and 0.22~0.24 nm/s, respectively, under a high vacuum of 1.5 × 10^−6^ Torr. The device active area is about 2 mm × 2 mm. After fabrication, the perovskite CsPbBr_3_ QD-LEDs were encapsulated in a glove box.

The electroluminescence (EL), current density-voltage-luminance (J-V-L) and external quantum efficiency (EQE) of the CsPbBr_3_ QD-LEDs were measured using the LQ-100R system (ENLI Technology) with 100 mm PTFE integrating sphere. The absorbance spectra of VB-FNPD and poly-TPD films were measured using an UV-visible spectrophotometer (V-770, JASCO). The photoluminescence (PL) spectra of the VB-FNPD films and CsPbBr_3_ QDs layers were measured using a fluorescence spectrophotometer (F-7000, Hitachi) with an excitation wavelength of 365 nm. The nanosecond time-resolved PL (TRPL) decay was analyzed by a commercial optical-microscope-based system (Fluoromax, Horiba). The wavelength, pulse duration, and repetition rate of the excitation for TRPL were 405 nm, 150 ps, and 20 MHz, respectively. The surface roughness was measured using an atomic force microscope (AFM, XE-70, Park Systems Corp).

## 3. Results and Discussion

**Figure 1:**
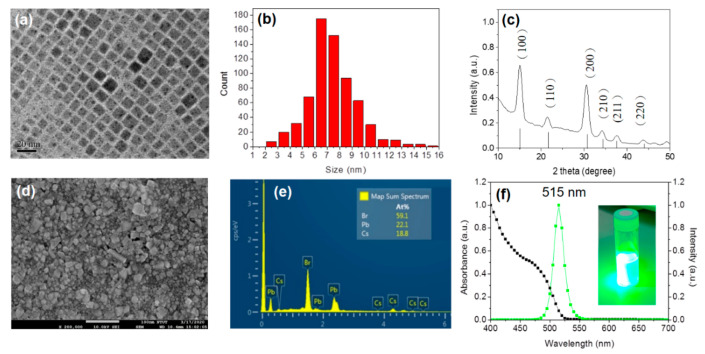
(**a**) TEM image and (**b**) size distribution of perovskite CsPbBr_3_ quantum dots (QDs). (**c**) XRD, (**d**) SEM image, (**e**) EDS specta, and (**f**) absorbance and PL spectra of CsPbBr_3_ QDs spun on glass substrates. The inset shows the color of CsPbBr_3_ QDs.

The QDs were synthesized via a typical hot-injection synthesis, an ice bath, and a purification treatment (see Materials and Methods for more details). The transmission electron microscopy (TEM) was used to check the morphologies of the QD preparing procedures. As shown in Figure 1a, our QDs are cubic shape and well dispersed in octane after removal of redundant precipitates via purification treatment, with an average size of 7~8 nm (Figure 1b). The cubic phase structure was further confirmed by the X-ray diffraction (XRD) patterns of CsPbBr_3_ QDs presented in Figure 1c. The peaks, (1,0,0), (1,1,0), (2,0,0), (2,1,0), (2,1,1), and (2,2,0), can be identified, which shows the cubic perovskite phase and is similar with a previous report [24]. Figure 1d illustrates a planar SEM image of the perovskite QD film which show highly dense and smooth morphology without aggregates. The energy dispersive spectroscopy (EDS) technique was used for qualitative analysis for our QD films, as shown in Figure 1e. The molar ratio of Cs:Pb:Br in our film was evaluated and very closed to 1:1:3. The PL and UV–vis absorption spectra (Figure 1f) of our CsPbBr_3_ QDs dispersed in octane show an emission peak is obtained at 515 nm and closed to its absorption edge, which corresponds to post studies [25,26,27]. It is worth mentioning that our QDs exhibits a bright green color, as shown in the inset of Figure 1f. These results demonstrate that our QDs have good crystalline and film qualities, which is highly beneficial for subsequent fabrication of QD-LED devices.

**Figure 2:**
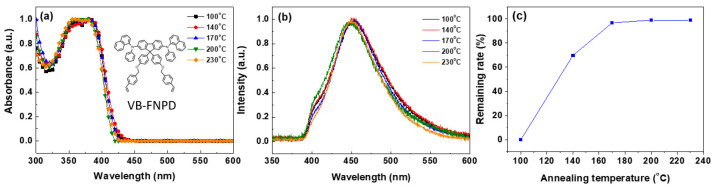
(**a**) Absorbance, (**b**) photoluminescence (PL), and (**c**) remaining rate of 9,9-Bis[4-[(4-ethenylphenyl)methoxy]phenyl]-N2,N7-di-1-naphthalenyl-N2,N7-diphenyl-9H-fluorene-2,7-diamine (VB-FNPD) films crosslinking at different temperatures. Remaining rate is obtained by measuring absorbance of VB-FNPD films before and after washing with cyclohexanone (CHO).

Figure 2a presents the absorbance spectra of VB-FNPD films crosslinking at different temperatures. It is shown that all the samples exhibit similar results in the range of 100~230 °C, which indicates that the thermally crosslinking reaction has ignorable effect on the absorbance of VB-FNPD films. Similar tendency is also shown in the PL curves of VB-FNPD films in Figure 2b. All the samples exhibit a peak at 449 nm, indicating a standard spectrum of the VB-FNPD film [22]. Similar results in absorbance and PL spectra are due to that the VB-FNPD films under different thermal polymerization consist of a same monomer. However, the AFM images of VB-FNPD films crosslinking at different temperatures show that the surface root-mean-square roughness (*R*_q_) increases with an increase of crosslinking temperature (Figure S1, Supporting Information), which can be ascribed to a larger VB-FNPD molecule via thermal crosslinking reaction and thus results in an increase of the TRPL decay-time as the crosslinking temperature increases from 170 to 230 °C [28]. Moreover, Figure 2c show the comparison of retention for VB-FNPD films crosslinking at different temperatures and corresponding samples after soaking in CHO. When crosslinking temperature is 100~140 °C, the intensity of absorbance has a larger difference in a range of 300~425 nm, implying scarcely VB-FNPD films remained (see Figure S2, Supporting Information). This phenomenon can be attributed to a weak crosslinking behavior of VB-FNPD at low temperature and thus results in the erosion by CHO. For an increase of crosslinking temperature to 170 °C, the intensity of absorbance improves, indicating the remaining VB-FNPD increases. As the crosslinking temperature continues to increase (170~230 °C), similar absorbance was obtained, which indicates the formation of stronger chemical bonds to prevent from the erosion by CHO.

**Figure 3:**
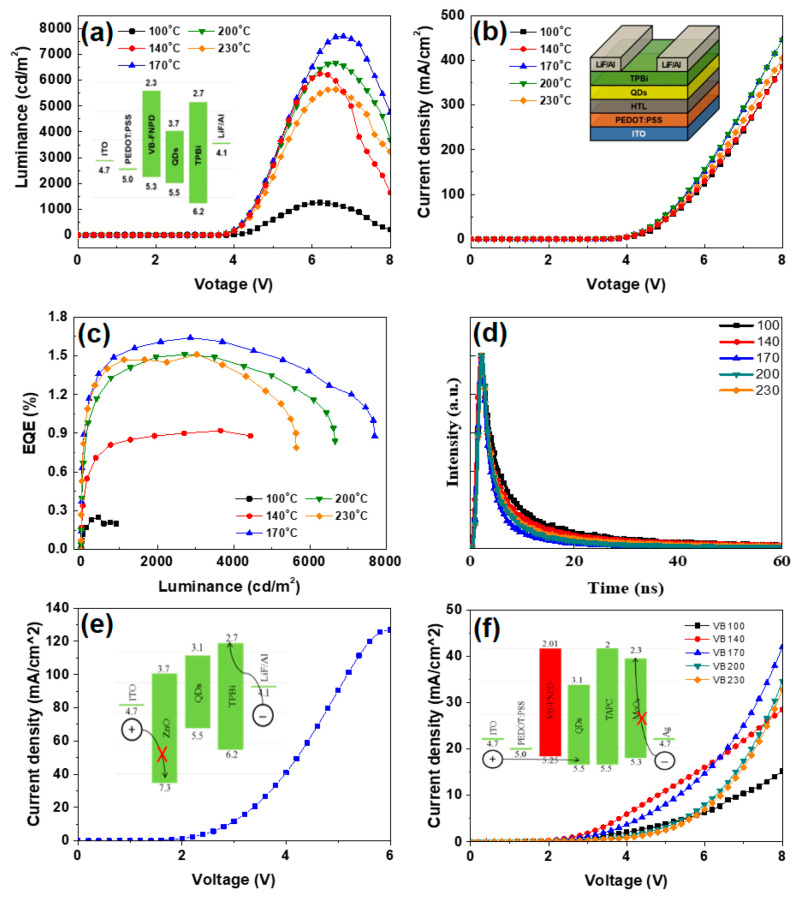
(**a**) Luminance-voltage (L-V), (**b**) current density-voltage (J-V), (**c**) external quantum efficiency (EQE) for CsPbBr_3_ QD-LED devices with VB-FNPD films crosslinking at different temperatures. The inset of (**a**) shows energy-band diagram of CsPbBr_3_ QD-LED. (**d**) TRPL decay profiles of perovskite CsPbBr_3_ QD layers on VB-FNPD films crosslinking at different temperatures. (**e**,**f**) show electron-only and hole-only devices for QD-LED devices. The insets of (**a**,**e**,**f**) show energy-band diagrams of the corresponding device structures. The inset of (**b**) shows the device schematic.

Figure 3 shows the device performance of the perovskite CsPbBr_3_ QD-LEDs with VB-FNPD HTLs crosslinking at different temperatures. In Figure 3a, the device with VB-FNPD HTL crosslinking at 170 °C has the highest L-V spectrum (7702 cd/m^2^ at 6.9 V). The similar tendency is also demonstrated in the J-V and EQE of the device. The device exhibits a turn-on voltage (*V*_T_) of 4.0 V, and the maximum EQE of 1.64%, as shown in Figure 3b,c, respectively. Of note, the above characterizations can be denoted that the thermally crosslinkable VB-FNPD HTL not only improves the device interface between the HIL and the AL emitter, but also revealed the balance between surface passivation and carrier injection [24]. The inset of Figure 3a shows the energy-band diagram of CsPbBr_3_ QD-LED. The HOMO and LUMO levels of VB-FNPD HTL and other layers can be referred to the results in [23,29]. It is noteworthy to point out that the similar trend of photoluminescence quantum yields (PLQYs) and the improvement of device performance can be simultaneously obtained. The summary of PLQY for perovskite CsPbBr_3_ QD layers on VB-FNPD films crosslinking at different temperatures is listed in Table 1. Figure 3d shows the TRPL decay profiles of the CsPbBr_3_ QDs layer on the VB-FNPD films crosslinking at different temperatures. The TRPL decay-time decreases as the annealed temperature increases from 100 to 170 °C. The 170 °C sample has the lowest decay times of 2.5 ns. It can be inferred that the non-radiative decay of the perovskite CsPbBr_3_ QDs layer is suppressed via the thermal crosslinking process of the VB-FNPD film, which is beneficial to increase the survival time and radioactive recombination probability of the excitons [30]. By contrast, the TRPL decay-time increases as the crosslinking temperature increases from 170 to 230 °C. This phenomenon is due to that the increased roughness results in more defects in the interface between the VB-FNPD and QD layers. Moreover, the electron-only and hole-only devices were also prepared to realize the effect of the crosslinking temperatures on the charge injection into CsPbBr_3_ QD films. For the hole-only device, the highest current is obviously increase in the device with VB-FNPD at 170 °C, also confirmed by the full QD-LED. Figure 3e,f demonstrate the efficiency of the electron injection is better than that of the hole injection, indicating that improved HTL is necessary for carrier balance. Overall, the similar trend in TRPL, PLQY, hole injection, and device performance can be contributed to the compete between surface roughness and polymerized degree.

**Figure 4:**
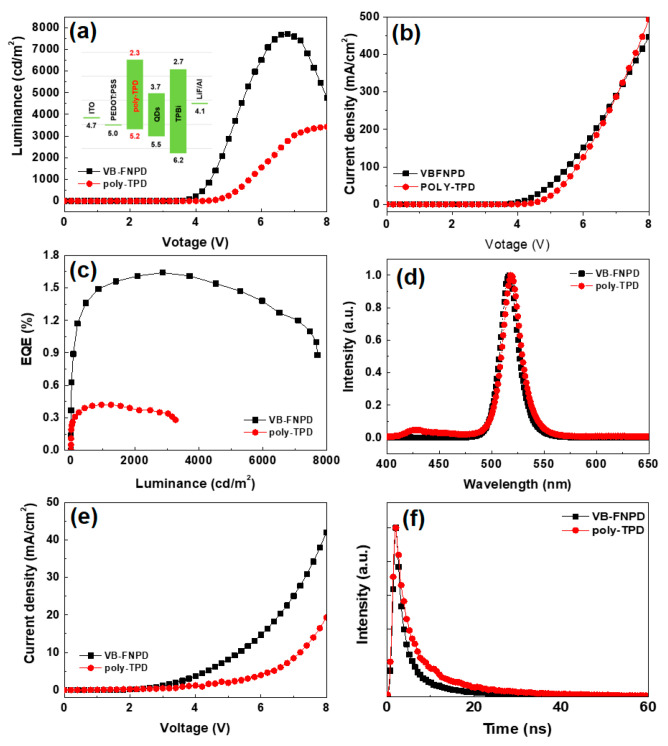
(**a**) L-V, (**b**) J-V, (**c**) EQE, (**d**) EL spectra, and (**e**) electron-only for CsPbBr_3_ QD-LED devices with VB-FNPD and poly-TPD HTLs. (**f**) TRPL decay profiles of perovskite CsPbBr_3_ QD layers spun on VB-FNPD and poly-TPD films.

In order to clarify the suitability of the thermally crosslinkable VB-FNPD as HTL of QD-LED in the remaining fabrication process, another common HTL material, i.e., poly-TPD was also discussed for comparison. Figure 4 shows the device performance of the perovskite CsPbBr_3_ QD-LEDs with VB-FNPD and poly-TPD HTLs. In Figure 4a, the device with VB-FNPD HTL has an L-V spectrum of 7702 cd/m^2^ (at 6.9 V), whereas that with poly-TPD HTL is 3426 cd/m^2^ (at 8 V). The similar tendency is also demonstrated in J-V and EQE of the device. The device with poly-TPD HTL exhibits a *V*_T_ of 4.1 V, and the maximum EQE of 0.42%, as shown in Figure 4a–c, respectively. The inset of Figure 4a shows the energy-band diagram of CsPbBr_3_ QD-LED with poly-TPD as HTL [31]. Overall, the above characterizations can be denoted that the perovskite CsPbBr_3_ QD-LEDs with VB-FNPD has better device performance than that with poly-TPD. In general, thermally crosslinkable VB-FNPD can improve the robustness of HTL and thus ameliorate the performance of QD-LEDs. Figure 4d illustrates the EL spectra of the perovskite CsPbBr_3_ QD layers on poly-TPD and VB-FNPD. It can be seen that both peaks have similar wavelength (515 nm), indicating a standard spectrum of the CsPbBr_3_ QDs [32]. Although the surface roughness of the poly-TPD films is better than that of VB-FNPD, the remaining rate of VB-FNPD washed by octane is almost 100% (Figures S3 and S4, Supporting Information), indicating that the VB-FNPD has a good resistance to octane (dispersant for CsPbBr_3_ QDs). It is important that the insolubility of the HTL layers can improve the interface and reduce defects. Therefore, the FWHM of the EL peak for poly-TPD and VB-FNPD were 22.3 and 21.7 nm, respectively. This result implies that the interface between the VB-FNPD and QD layer has a better contact. Moreover, the shoulder EL peak of 425 nm is the carrier recombination in the poly-TPD layer (see Figure S4, Supporting Information) due to poor carrier balance. Figure 4e presents the hole-only devices with poly-TPD and VB-FNPD HTLs. The obvious hole-injection efficiency can be found in the VB-FNPD based device. Figure 4f shows the TRPL decay profiles of the CsPbBr_3_ QDs layer on poly-TPD and VB-FNPD. The QDs/VB-FNPD has a lower decay-time than QDs/poly-TPD and this behavior corresponds to the results of EL intensity (i.e., a lower decay-time indicates a stronger EL intensity). Overall, the thermally crosslinkable VB-FNPD is found to provide a better resistance to the QD dispersant (i.e., octane), implying a potential candidate as HTL of QD-LED in the remaining fabrication process.

## 4. Conclusions

This study has examined the effect of thermally crosslinkable VB-FNPD as HTL of QD-LEDs. It has been shown that the film robustness of HTL is improved by the formation of strong chemical bonds through crosslinking behavior and thus it can resist the erosion from the organic solvent in the fabrication process. In general, the results confirm that the proposed thermally crosslinkable VB-FNPD as HTL is a candidate for the development to QD-LEDs.

## Figures and Tables

**Table 1:** Summary of photoluminescence quantum yields (PLQY) values of VB-FNPD films annealed at different temperatures.

	100 °C	140 °C	170 °C	200 °C	230 °C	poly-TPD
PLQY (%)	18	18.5	19	18.5	18.3	10

## Supplementary Materials

The following are available online at https://www.mdpi.com/2073-4360/12/10/2243/s1, Figure S1: Surface AFM images of VB-FNPD films crosslinking at different temperature, Figure S2: Comparison of absorbance for VB-FNPD films crosslinking at different temperature and corresponding samples before and after soaking in CHO, Figure S3: Comparison of washing effect (in octane) of absorbance for (a) poly-TPD and (b) VB-FNPD films, Figure S4: AFM image and PL spectrum of a Poly-TPD film.
