# Supplementary material for: Using Thermally Crosslinkable Hole Transporting Layer to Improve Interface Characteristics for Perovskite CsPbBr3 Quantum-Dot Light-Emitting Diodes
Source: Polymers (Basel). 2020 Sep 29;12(10):2243. doi: 10.3390/polym12102243 (PMC7601921; doi:10.3390/polym12102243)
Supplement: Supplementary file 1 [file polymers-12-02243-s001.pdf]

Supplementary Materials

Using Thermally Crosslinkable Hole Transporting Layer to Improve Interface Characteristics for Perovskite CsPbBr3 Quantum-Dot Light-Emitting Diodes

Chun-Cheng Lin [1], Shao-Yang Yeh [2], Wei-Lun Huang [3], You-Xun Xu [3], Yan-Siang Huang [3], Tzu-Hung Yeh [4], Ching-Ho Tien [2], Lung-Chien Chen [2,*] and Zong-Liang Tseng [3,4,*]

[1] Department of Mathematic and Physical Sciences, General Education, R.O.C. Air Force Academy, Kaohsiung 820008, Taiwan; cclincafa@gmail.com

[2] Department of Electro-optical Engineering, National Taipei University of Technology, Taipei 10608, Taiwan; iamarvin0811@gmail.com (S.-Y.Y.); chtien@mail.ntut.edu.tw (C.-H.T.)

[3] Department of Electronic Engineering, Ming Chi University of Technology, New Taipei City 243303, Taiwan; a88061446@gmail.com (W.-L.H.); sasa220404373@gmail.com (Y.-X.X.); jim824645@gmail.com (Y.-S.H.)

[4] Organic Electronics Research Center, Ming Chi University of Technology, New Taipei City 243303, Taiwan; d10502004@mail.ntust.edu.tw

* Correspondence: ocean@ntut.edu.tw (L.-C.C.); zltseng@mail.mcut.edu.tw (Z.-L.T.)

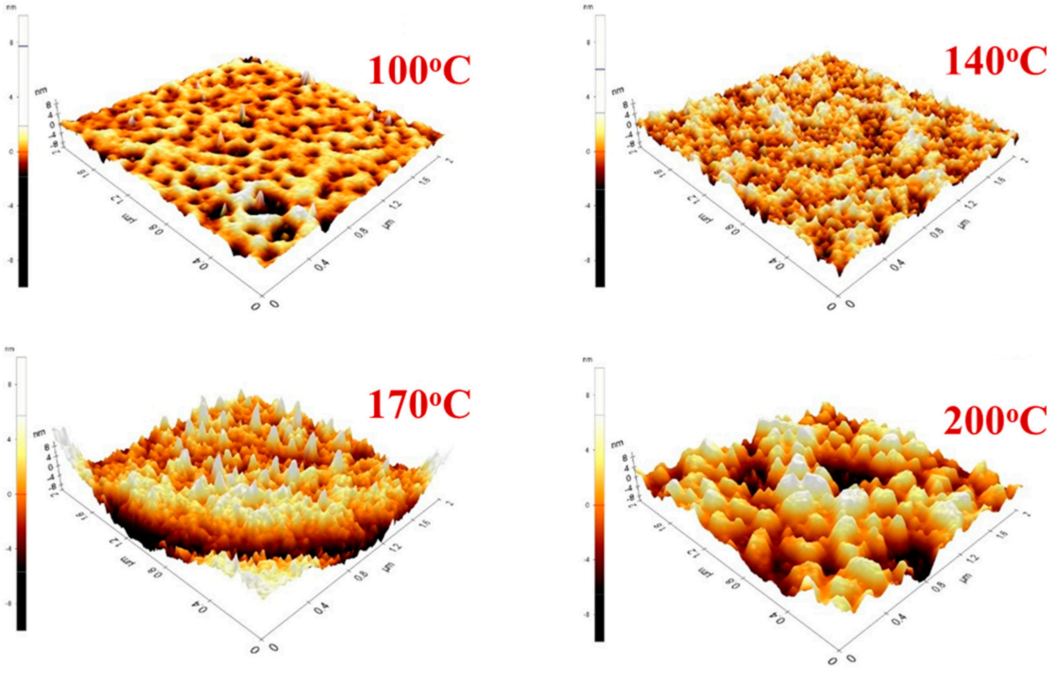

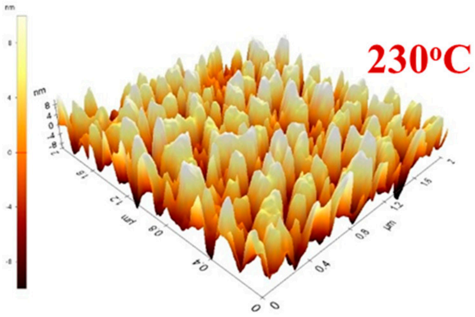

Temperature (°C)	R_q (nm)
100	0.944
140	1.437
170	2.953
200	3.345
230	5.771

Figure S1. Surface AFM images of VB-FNPD films crosslinking at different temperature.

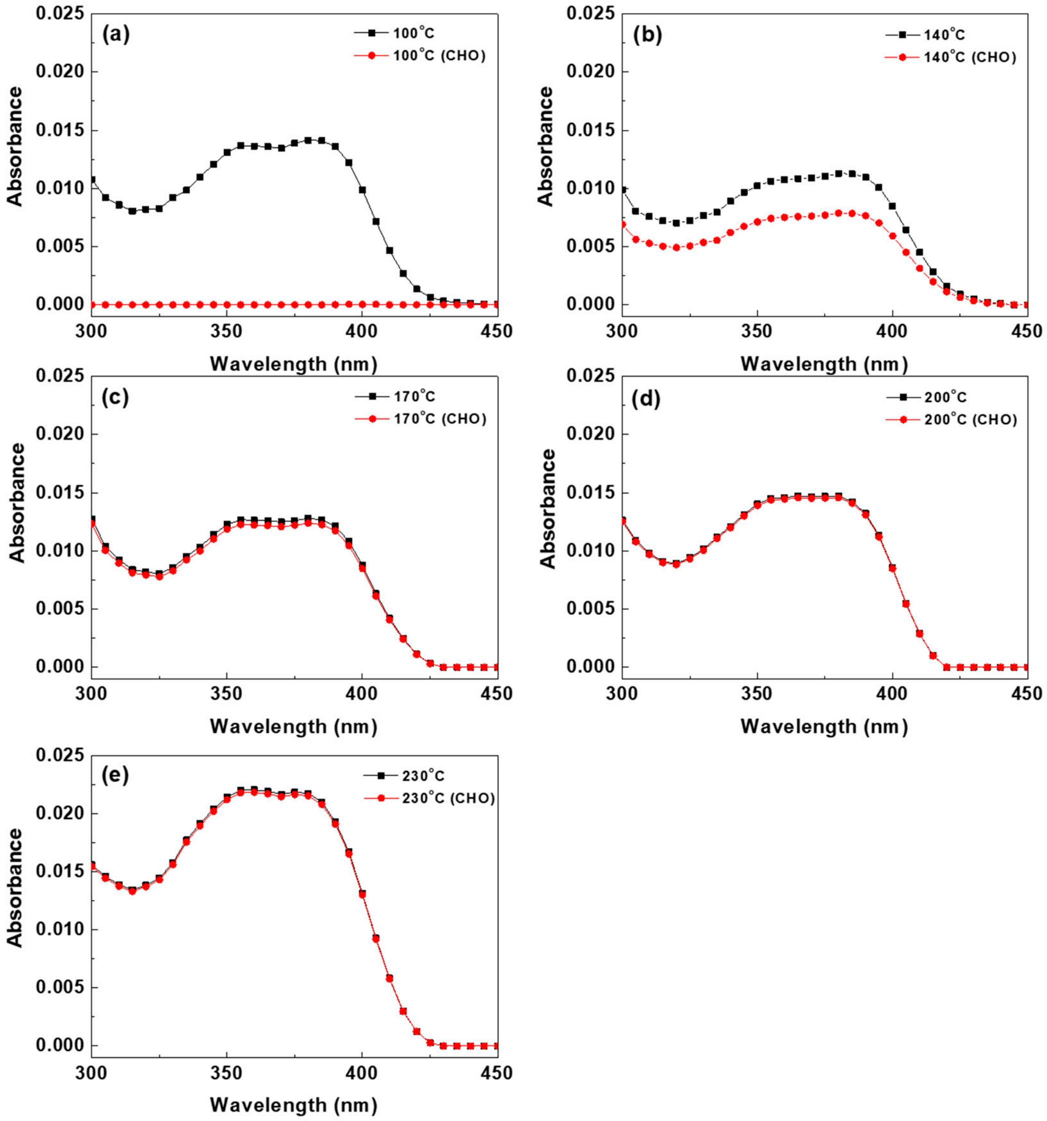

Figure S2. Comparison of absorbance for VB-FNPD films crosslinking at different temperature and corresponding samples before and after soaking in CHO.

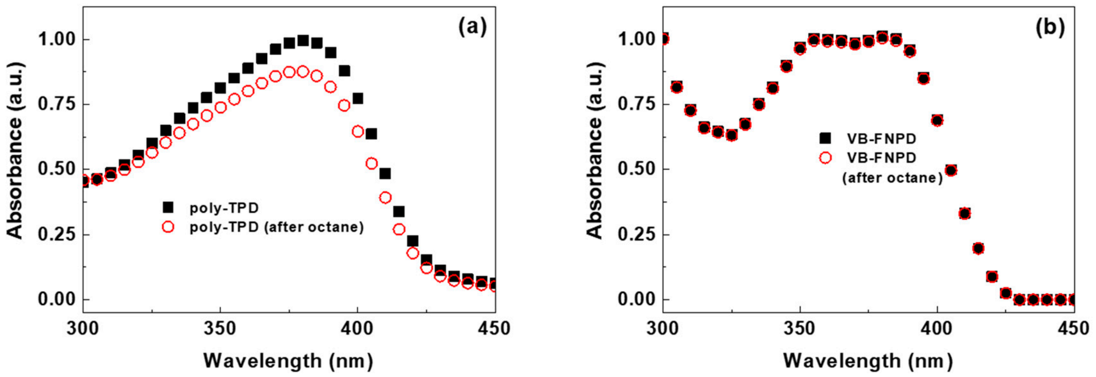

Figure S3. Comparison of washing effect (in octane) of absorbance for (a) poly-TPD and (b) VB-FNPD films.

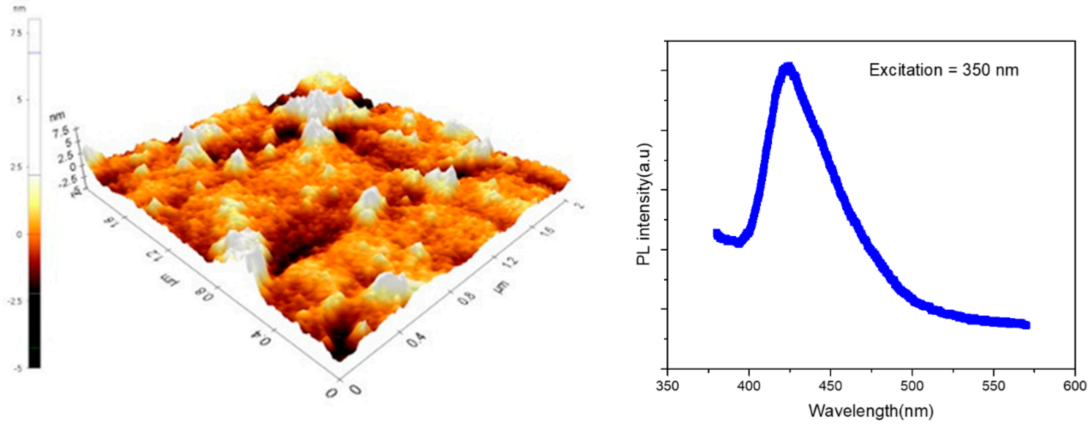

Figure S4. AFM image and PL spectrum of a Poly-TPD film.
